# Fire ants: What do rural and urban areas show us about occurrence, diversity, and ancestral state reconstruction?

**DOI:** 10.1590/1678-4685-GMB-2021-0120

**Published:** 2022-03-07

**Authors:** Manuela de O. Ramalho, Leonardo Menino, Rodrigo F. Souza, Débora Y. Kayano, Juliana M. C. Alves, Ricardo Harakava, Victor H. Nagatani, Otávio G. M. Silva, Odair C. Bueno, Maria S. C. Morini

**Affiliations:** 1 Cornell University, Department of Entomology, Ithaca, USA.; 2 Universidade de Mogi das Cruzes, Mogi das Cruzes, SP, Brazil.; 3 Secretaria de Agricultura e Abastecimento, Instituto Biológico, Laboratório de Bioquímica Fitopatológica, São Paulo, SP, Brazil.; 4 Museu Paraense Emilio Goeldi, Coordenação de Ciências da Terra e Ecologia, Belém, PA, Brazil.; 5 Universidade Estadual Paulista (UNESP), Instituto de Biociências, Departamento de Biologia Geral e Aplicada, Rio Claro, SP, Brazil.

**Keywords:** Biodiversity, haplotype diversity, DNA barcoding, mtDNA

## Abstract

In South America, *Solenopsis saevissima* and *S. invicta* are the most common fire ants. Nests are founded in areas under anthropic interference like urban or rural areas, but *S. invicta* is found preferentially in those with the greatest anthropic interference. However, we do not know the rates at which they exist in anthropized areas next to high density of native vegetation. Areas with 60 to 90% of native Atlantic Forest were selected to verify the occurrence of both species in rural and urban areas. We investigated the molecular diversity and applied the reconstruction of the ancestral state analysis for each species. A total of 186 nests were analyzed and we found that the two species had the same proportion in the urban area. However, *S. saevissima* had a higher rate of prevalence in the rural area, in addition to having a greater number of haplotypes and ancestry associated with this type of habitat for the region. *S. invicta* had the same number of haplotypes in both rural and urban regions, and less haplotypic diversity. We conclude that *S. saevissima* is a species typically associated with rural areas and *S. invicta*, although present, is not dominant in urban areas.

## Introduction

*Solenopsis* (Myrmicinae: Solenopsidini) has 196 species, in addition to 22 subspecies (Bolton 2021), with a widespread distribution. In the Neotropical region, there are a total of 101 species; 43 of these species are found in Brazil and 25 of them are found in the state of São Paulo (AntWeb, 2021). Species-level identification is difficult (Trager, 1991; Pacheco et al., 2013), especially in the Neotropical Region which has high diversity (Pitts et al., 2018). There are species characterized by workers of small size and, monomorphic that form small colonies and can exhibit a lestobiotic lifestyle, known as thief ants (Pacheco *et al.,* 2013). Others are known as fire ants, which include species with larger workers, polymorphic and populous colonies (Trager, 1991).

Fire ants are found in South America (Pitts et al., 2018), where they exhibit omnivorous, opportunistic and aggressive behavior (Pitts, McHugh and Ross, 2005). In addition, they display variation in social structure (Tschinkel, 2006) and in larvae morphology (Pitts *et al.,* 2018). In this group, we still find social parasitic species (e.g., *S. daguerrei* and *S. hostilis*) (Pitts *et al.,* 2018). The nests are founded, especially, in open and sunny areas, such as pastures (Pacheco and Vasconcelos, 2007; Rabello et al., 2018), crops (Lunz et al., 2009) and urban (Ulloa-Chacon, 2003; Pacheco and Vasconcelos, 2007; Zeringóta et al., 2014). Fire ants cause damage to biodiversity (Dejean et al., 2015), crops (Chan and Guenard, 2020) and also health problems for people who are allergic to the venom (Haddad Junior and Larsson, 2015). Farmers estimate losses of 10% to 80% in production due to fire ants, especially related to *S. invicta* (Chan and Guenard, 2020). In addition to damage to agriculture, the USA and China report impacts of *S. invicta* on civil construction and public health sectors (Banks et al., 1990; Wang et al., 2019). In Brazil, there is little knowledge about the impact caused by fire ants on agriculture, however there are records of large infestations associated with health problems, mainly due to allergies (Fernandes et al., 2016).

In South America, *S. saevissima* and *S. invicta* are the most common fire ants (Fowler et al., 1990; Tschinkel, 2006; Pitts et al., 2018). *S. saevissima* is distributed throughout the Brazilian coast, including the Amazon region. *S. invicta* is largely in the Pantanal region, close to the Paraguay River. This area is composed of savannas and seasonally inundated wetlands (Pitts *et al.,* 2018). Both species are found in rural (e.g., crops) and urban areas (Fowler, Bernardi and di Romagnano, 1990; Dejean et al., 2015). However, the area of occurrence of *S. invicta* is expanding to exclusive sites of *S. saevissima* (see Pitts *et al.,* 2005, Martins et al., 2014; Fox et al., 2012; Souza et al., 2014).

Morphologically the nests are similar both on the outside (Penick and Tschinkel, 2008; Zeringóta et al., 2014) and on the inside, where they consist of labyrinths and galleries about 1 to 1.5 m deep. These galleries are used for protection, movement of workers in search for food, storage of resources and garbage disposal (Penick and Tschinkel, 2008; Wilder et al., 2013). Both species are aggressive (Roux et al., 2013; Lai et al., 2015) and, although they depend on practically the same resources, foraging behavior occurs in different periods. *S. saevissima* is more nocturnal (Orivel and Dejean, 2002; Dejean *et al.,* 2015) and *S. invicta* has diurnal habits (Porter and Tschinkel, 1987).

The use of land for different purposes alters the landscape structure, as e.g., by loss of biotic and abiotic resources. That can affect ant communities (Crist, 2009), benefiting some species at the expense of others (Schoereder et al., 2004). The Atlantic Forest biome has been hurt by fragmentation (Joly et al., 2014), which is mostly related to anthropogenic activities (Tabarelli et al., 2005; Ribeiro et al., 2009). Therefore, we seek in this study to investigate the occurrence of *S. saevissima* and *S. invicta* in rural and urban areas interspersed with fragments of the Atlantic Forest. Even though *S. invicta* is associated with more ecologically disturbed environments, especially related to human-associated habitats (King and Tschinkel, 2008; Bertelsmeier et al., 2015), we expect to find fewer nests in urban areas when compared to *S. saevissima* because the region we chose to study has many fragments of conserved Atlantic Forest (Sartorello, 2018). This can be a barrier to the dispersion of the species. In addition, we seek to identify the molecular diversity of *S. saevissima* and *S. invicta* and apply the reconstruction of the ancestral state analysis for each species in order to fill the knowledge gap regarding the origin of the dispersion (urban or rural) for each species.

## Material and Methods

### Collection areas

The collections were carried out in areas belonging to the Atlantic Domain of the Southeast region of Brazil (Fiaschi and Pirani, 2009; Colombo and Joly, 2010). The collections were carried out between September 2015 and March 2017, on sunny days and with a minimum rain interval of one week. In the urban and rural areas (n = 21 locations in each area; Table S1) we carry out a linear transect (total = 12 km^2^), where nests were collected every 20 meters. Classification of rural and urban areas is in accordance with the Brazilian Institute of Geography and Statistics (IBGE, 2020). Thus, we considered rural areas as places destined for agricultural or livestock activities; and urban area where there is the presence of city infrastructure (e.g., pavement, rainwater channeling, water supply, sanitary sewer system and public lighting network).

Entire nests were sampled from the surface to a depth of 5 cm, using a gardening shovel (see geographical coordinates in Table S1). The collected content was placed in a plastic pot (5 L) previously coated with teflon. In the laboratory, the ants were separated from the soil using the drip technique (Bueno, 2017). To standardize this collection, the entire procedure was performed by the same person. Then the ants were placed in 95% ethanol and stored in a -20 ºC freezer at the Alto Tietê Myrmecology Laboratory of University of Mogi das Cruzes, São Paulo, Brazil. Field capture and collections were authorized by the Chico Mendes Institute for Biodiversity Conservation, Brazil (ICMBio / SISBIO permits n. 66500). For the following analyses, 186 nests were selected (rural area: 93; urban area: 93; Table S1). 

### Species identification

Morphological identification was performed using three major workers from each nest (see Pitts et al., 2018). For better visualization of the morphological characters, Scanning Electron Microscopy (SEM) images were performed, specifically from the frontal view of the head, lateral view of the mesosome, and view of the posterior portion of the post-petiole of the specimens. High-resolution images in front view were taken in the multi-focus image overlay system, with the AutoMontage® program and a Leica M205C® stereomicroscope coupled to a Leica DFC 295® camera. 

We performed molecular identification in all nests (Figure S1). The specimens (n = 3 for each nest) were also identified using the DNA Barcode technique, which uses a stretch of mtDNA - COI and compares with sequences deposited in a database, such as GenBank (https://www.ncbi.nlm.nih.gov/genbank/) and BOLDSYSTEM (http://www.boldsystems.org/) (Hebert et al., 2003; Ratnasingham and Hebert, 2007). The total DNA of each organism was extracted separately, following the protocol adapted from Martins et al. (2014). The total DNA of each specimen was used to generate an approximately 920 bp fragment of Cytochrome Oxidase I - COI, using the CIJ and DDS primers described by Ahrens et al. (2005). The sequencing reactions were performed with the reagent BigDye® Terminator v3.1 Cycle Sequencing Kit (Life Technologies - Applied Biosystems) and the equipment used was ABI 3730 DNA Analyzer (Life Technologies - Applied Biosystems). We edited the obtained sequences using the software BIOEDIT (Hall, 1999) and MUSCLE 3.6 (Edgar, 2004) and compared with the GenBank databases (https://www.ncbi.nlm.nih.gov/genbank/) and BOLDSYSTEM (http://www.boldsystems.org/). 

### Data analysis

The number of nests for each species and area was compared using Mann-Whitney test with a significance level of 5% (BioEstat 5.0 program; Ayres and Ayres Junior, 2000). The identification of specific mtDNA - COI haplotypes, as well as haplotypic (h) and nucleotide (π) diversity were performed using the DnaSP 4.9 software (Rozas et al., 2003). The haplotype network was created using the Network 4.5 software (fluxus-enginnering.com), using the parameter Median-Joining (Bandelt et al., 1999). 

We aligned the sequences obtained with ClustalW (Higgins et al., 1992) from the BioEdit software (Hall, 1999). Next, we used the Model Finder software (Kalyaanamoorthy et al., 2017) to choose the best molecular evolution model according to AICc for each species, being TPM2u + F + G4 and TPM2u + F + I the best model for *S. saevissima* and *S. invicta*, respectively. Subsequently, we inferred the phylogenetic tree through the Maximum likelihood method with 1000 bootstrap replicates for each species in the online IQ tree software (Nguyen et al., 2015; Trifinopoulos et al., 2016), using the sequence of *S. interrupta* (Cod. Genbank AY950727) as an outgroup. The Ape and Phytools packages (Paradis et al., 2004; Maintainer and Revell, 2020) of software R (R Core Team, 2021) were used to reconstruct ancestral character states (Reconstruction of Ancestral State - SRA) from the original habitat of these *Solenopsis* species for the region. To this end, we assigned each habitat of origin (urban or rural) to each tip of our topologies. The ‘equal rates’ (ER) model and the ‘all different rates’ (ARD) model were compared with the likelihood ratio test (LRT) (Pagel, 1994; Schluter et al., 1997) to determine which model best fits for our date. The probability distribution of the states was calculated running 10000 generations of MCMC, sampling every 100 generations (Huelsenbeck et al., 2003). Through SIMMAP function (Bollback, 2006) we generated 100 maps of stochastic characters from our data set, which were summarized considering the number of changes, the proportion of time spent in each urban or rural state and the latest probabilities that each internal node is in each state, under the best model. 

## Results

The morphological identification shows us that workers of *S. saevissima* have the head subquadrate to weakly ovate, lack of median frontal streak, complete mandibular costulae, mesonotum is weakly convex in lateral view, and post-petiole in posterior view is higher than wide, with transversely rugose sculpture only in the lower portion and surface in the upper portion is smooth shiny (Figure S1). In the workers of *S. invicta*, we observed the head subquadrate to weakly cordate, presence of the median frontal streak, mandibular costulae absent medially, mesonotum convex in lateral view, post-petiole is wider than high in posterior view with sculpture transversely rugose to punctate-rugose covering most of the view (Figure S2). *Solenopsis saevissima* can be distinguished from *S*. *invicta*, and both from the others fire ant’s species, by these diagnostics characters, as described by Pitts et al. (2018).

**Figure 1 -:**
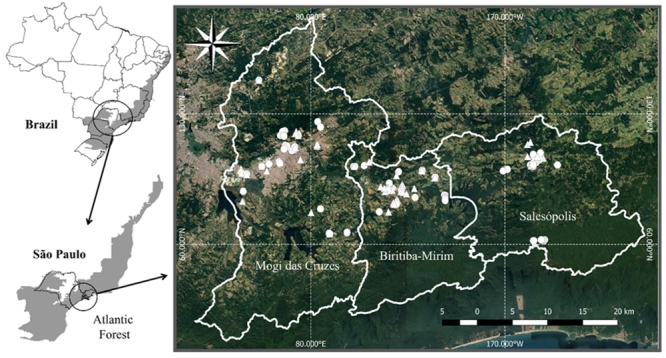
Geographic location of the counties and collection sites of *Solenopsis* nests. Circle: *S. saevissima* (n= 103); triangle: *S. invicta* nests (n= 83).

Molecular sequencing confirmed the results of the morphological identification. The mtDNA sequences of the collected specimens were compared with those deposited in the GenBank database. Thus, our study found 103 nests of *S. saevissima* and 83 nests of *S. invicta* (Figure 1; Table S1). Our sequences showed 98% to 100% similarities with other *Solenopsis* spp. already identified in the database. All details of the identifications with the respective access codes were summarized in Table S1. Regarding the haplotype diversity recovered in the present study, *S. saevissima* has the greatest haplotype diversity compared to *S. invicta* (Table 1).

**Table 1 -:** Number of haplotypes and haplotype diversity found in each species analyzed.

	*Solenopsis saevissima* (n=103 nests)	*Solenopsis invicta* (n=83 nests)
	Number haplotypes	Number haplotypes
Rural	13	6
Urban	6	6
	Haplotype diversity	Haplotype diversity
Rural	0.7998	0.8367
Urban	0.6185	0.3521
General	0.9105	0.6985
	Final length of sequences	
	~792 base pairs (bp)	

**Figure 2 -:**
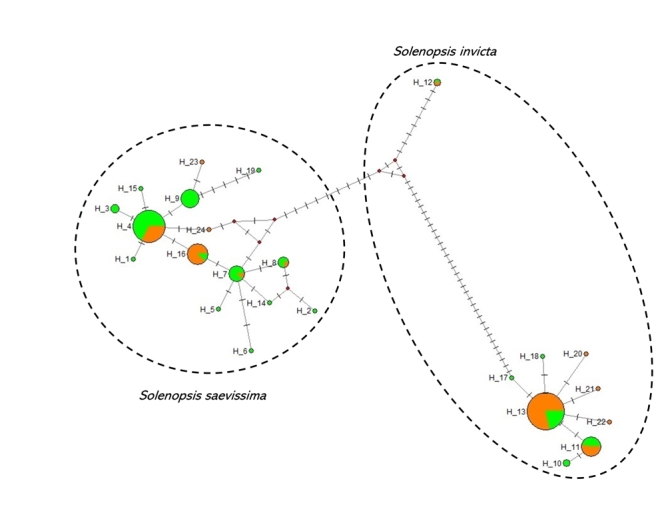
Network of haplotypes of *Solenopsis saevissima* and *Solenopsis invicta*. The habitat of the sample was highlighted with the colors green and orange corresponding to the rural and urban habitats, respectively. Dotted circles indicate the delimitation of each species. The red point was added by the program as hypothetical haplotype.

The network analysis facilitates the visualization of the two distinct species and their haplotypes explored in this study at different habitats (Figure 2). *Solenopsis saevissima* has a higher number of haplotypes associated with the rural habitat, while *S. invicta* has the same occurrence of haplotypes in both rural and urban habitat. In addition, our data identified 15 haplotypes of *S. saevissima* in total. The majority of these haplotypes were found exclusively in the urban or rural habitat. However, there were also the presence of some haplotypes occurring in both urban and rural habitat, i.e., H_4, H_16, H_7 and H_8 (Figure 2). For *S. invicta* we recovered nine distinct haplotypes, three of which occurred in both urban and rural habitat (H_11, H_12 and H_13). The remaining haplotypes were found to be exclusive to a particular habitat, as can be seen in Figure 2. Still on the network analysis, despite the fact that H_12 is classified as *S. invicta* and was found as in urban and rural habitat, it still presented quite distinct of the other *S. invicta* haplotypes recovered in the present study. This H_12 haplotype has already been identified in previous studies in *S. invicta* in Mississippi, USA (access code EU352608), as well as in Argentina (access code JN808817). More studies targeting different genes may contribute more to understanding the evolutionary history of *Solenopsis*, in particular about this H_12 haplotype. 

**Figure 3 -:**
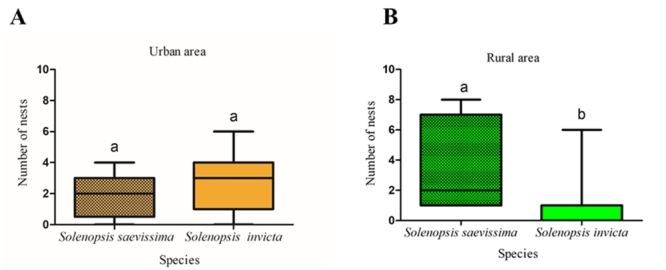
Comparison of the number of nests in urban (A) and rural (B) populations of *Solenopsis saevissima* and *Solenopsis invicta*. The lines inside each bar represent the median. Different letters represent statistically significant differences (Mann-Whitney test, p <0.05).

We then analyzed whether *S. invicta* and *S. saevissima* occur at different frequencies in urban and rural habitats. In the rural area *S. invicta* was registered less occurrence of nests (U = 89.50; Z (u) = 3.29; p = 0.0005; Figure 3A), when compared to *S. saevissima*. In the urban area, these species have the same occurrence of nests (U = 167.00; Z (u) = 1.3458; p = 0.1784; Figure 3B). 

In order to proceed with the ancestral state reconstruction analyses, we first tested the best evolutionary model for our data. Therefore, likelihood ratio testing (LRT) showed that the best model that fits our data was the ARD (all rates different). This ARD model considers that the rate transitions in each state (rural or urban) are different, compared to the ER model (equal rates) (see Table S2).

**Figure 4 -:**
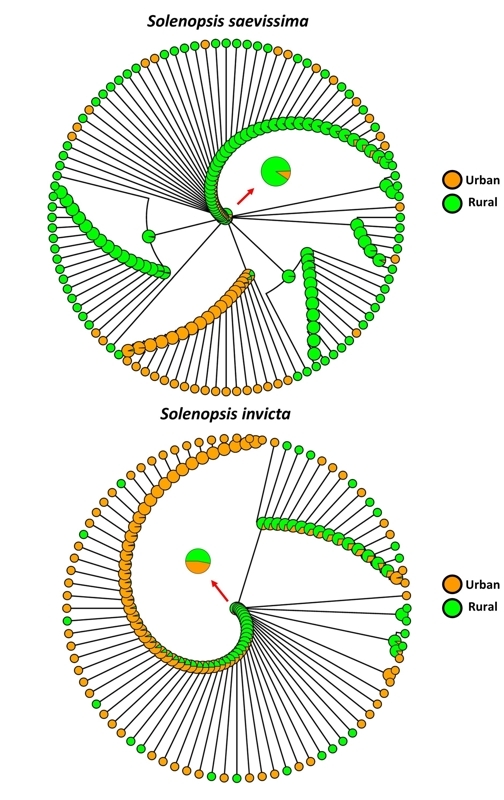
Summary of all stochastic character maps for ancestral state reconstruction of the habitat of *Solenopsis saevissima* and *Solenopsis invicta* for the studied region. Red arrows highlight the probability of the state of the ancestor of each species. Model = all rates different (ARD).

Once chosen the best evolutionary model, we then tested the probability of the habitat of origin of each species with ancestral state reconstruction (ASR) analysis. Our ASR results shows that the ancestor of *S. saevissima* for the studied region has a 90% probability of having a rural origin (90% rural and 10% urban), while *S. invicta* 53% rural and 47% urban. The red arrows in Figure 4 highlight these ancestral state probabilities for each species (see Figures S3 and S4 for the entire names of the tips). In addition, for *S. saevissima* and *S. invicta* there were multiple transitions from rural to urban environment with a special emphasis on a lineage of *S. saevissima* that once acquired the status of urban, remained urban practically throughout the clade, with only a few exceptions. This shows that there may be a specialization of this lineage of *S. saevissima* for urban habitat, despite the great majority of the recovered diversity being associated with the rural environment (Figure 4). The analysis of ancestral state reconstruction (ASR) for the habitat of *S. invicta* for the region shows under the lens of phylogeny that in addition to having multiple transitions between rural and urban environments, there is no specialization of lineage for these habitats. However*, S. invicta* is overall more associated with the urban environment (Figure 4).

## Discussion

In this study we analyzed the occurrence of two species of fire ants (*S. saevissima* and *S. invicta*) in urban and rural habitats next to fragments of the Atlantic Forest (Neotropical region) (see Figure 1 and Table S1). The present study evaluated the frequency of occurrence, haplotype diversity and also brought insights about the habitat of origin of each fire ant species for this region. We used morphological characters suggested by Pitts et al. (2018) and DNA Barcode technique for species diagnosis, because *Solenopsis* species identification is complex due to the high degree of polymorphism of workers (Pitts *et al.,* 2005, 2018), and presence of hybridization (Stolle et al., 2021). Our results suggest that the use of both methods is crucial for the identification of *S. saevissima* and *S. invicta.*

Our results show that *S. saevissima* and *S. invicta* are present in both areas, however, we show that the frequency of occurrence is not the same. The non-dominance of *S. invicta* is likely related to the characteristics of urbanization [e.g., the presence of more native vegetation and grass on the soil surface (see Kafle et al., 2009) or to abiotic factors, e.g., temperature and rainfall (Korzukhin et al., 2001; Morrison et al., 2004; Byeon et al., 2020; Chen et al., 2020), which should be more favorable to *S. saevissima*. Additionally, we suggest that in urban areas, the populations of *S. saevissima* must be reduced due to the competitive, strong adaptive and reproductive capacity of *S. invicta* to explore resources and colonize diverse habitats (Fowler, Bernardi and di Romagnano, 1990; Ross and Shoemaker, 2008; LeBrun, Plowes and Gilbert, 2012). The fire ant social organization could also indicate the success of the species, as polygynous colonies have a high number of individuals and queens has a lower dispersal capacity when compared to monogynous colonies (Macom and Porter, 1996; Deheer et al., 1999; Stolle et al., 2021). However, in previous studies we investigated this component by sequencing the *GP-9* gene with samples from the same nests in the present study. Our results confirmed that all colonies of the two species were monogynous (see Alves et al., 2018; Souza, 2019).

In this study, we observed populations of *S. saevissima* with darker specimens, which is a variant registered in Brazil between Goiás and Bahia, including São Paulo State (Pitts et al., 2018). *Solenopsis invicta* is characteristic of the northern region of Porto Velho (Rondônia State, Brazil) and Cuiabá (Mato Grosso State, Brazil) (Pitts *et al.,* 2018), and in neighboring countries like Argentina, Bolivia, Paraguay, and Uruguay (Pitts, McHugh and Ross, 2005; Ross *et al.,* 2007). In the state of São Paulo (southeastern Brazil) the species is associated with the Cerrado patches (Fowler et al., 1990), with expansion of territory to areas under Atlantic Forest dominance (Martins et al., 2014; Souza et al., 2014). In addition to agriculture (Pacheco et al., 2013, 2017), urban growth contributes greatly to the expansion of the foraging territory of the populations of *S. invicta*, increasing its range of distribution (Plowes et al., 2007). Our study areas include a region with accelerated urban growth (Lima and Magaña Rueda, 2018) and, at the same time, many conserved remnants of the Atlantic (Torres et al., 2019). *Solenopsis invicta* does not affect native ant communities in more preserved forest remnants, suggesting that their competitive dominance occurs largely in disturbed habitats (King and Porter, 2007).

When native habitats are disturbed and/or fragmented, the introduction and establishment of invasive species is facilitated (Sakai et al., 2001; Holway et al., 2002). As the native vegetation of the Atlantic Forest has been strongly fragmented (Marques, 2020), the environment is favorable for *S. invicta* to expand its territory. Analyses of genetic diversity of populations of *S. invicta* (Souza, 2019) show that the species is undergoing an expansion process in the same region of our study.

In places where native vegetation is dominant in the landscape, as is the case in rural areas (e.g., in the vicinity of agricultural properties and dirt roads), *S. saevissima* was the most abundant species. In addition, our results on haplotypic diversity show that *S. saevissima* is the more diverse, suggesting that it is the species native to the region, as had been discussed by Pitts et al. (2005). Also, high haplotype diversity values of this species corroborate data from Ross et al. (2010). This evidence corroborates with the observed of *S. saevissima* being more likely to be found in rural sampling sites and coming from an older population.

In contrast, *S. invicta* recovered in this study showed lower genetic diversity, which is consistent with previous studies (Ahrens et al., 2005). Additionally, one haplotype appears to be the most frequent and dominant in the region (H_13), with several additional haplotypes having recent ancestry from the main haplotype. These data suggest that *S. invicta* is expanding to the region, but with a pattern consistent with a bottleneck introduction and its subsequent spread. A similar pattern was observed for *S. invicta* populations in Taiwan, and USA where studies also found evidence of recent introduction associated with molecular signatures of genetic bottlenecks (Shoemaker et al., 2006; Yang et al., 2008,). 

In addition, our reconstruction of the ancestral state results shows that *S. saevissima* is very likely to have a rural ancestor, which is expected since it is native to the region. And even with all the associated diversity, *S. saevissima* in general continues to present a greater probability of being associated with rural habitat (Lunz et al., 2009; Martin et al., 2011), except for a lineage that evolved for urban. In contrast, *S. invicta* presented a probability of 53% rural and 47% urban of its habitat ancestral state. However, although one lineage specialized in rural first, another lineage specialized in urban, and this seems to be the most common. These data explain in light of the evolutionary process of ancestry why *S. saevissima* seems to be more propitiate to rural habitat and *S. invicta* seems to be more successful in an urban environment, thus corroborating several other studies prior to this one (Tsutsui and Suarez, 2003; Gusmão et al., 2010; Wetterer and Davis, 2010). Although our data advance the knowledge about the diversity and origin of these species for the region, we need to recognize that the present study only considered the mitochondrial lineage. Therefore, future studies should incorporate other genetic markers, especially nuclear genes.

## Conclusions

Our results show that the most common species of fire ants in Brazil are not found in equal amounts in urban and rural habitats. We show that *S. saevissima* is more characteristic of rural environments with greater haplotypic diversity. In the urban environment, *S. invicta* is not characterized as the dominant species, dividing the foraging territory with *S. saevissima*. Our work is the first to investigate the ancestral state of urban and rural habitats of both *S. saevissima* and *S. invicta* species in the Atlantic Forest Dominion region in the evolutionary context. Thus, the present work adds important information that serves as a subsidy for the protection of *S. saevissima*, which is the species native to the region, according to our results. The maintenance of vegetation in urban areas can be a good mechanism to prevent the advancement of *S. invicta* in the region. As the study areas belong to the Brazilian Atlantic Domain, our results, in a more comprehensive way, can help public policy programs aimed at the conservation of this biome. 

## Supplementary material

The following online material is available for this article:

Table S1 - Samples of *Solenopsis saevissima* and *Solenopsis invicta* collected in the present study.

Table S2 - Likelihood ratio test (LRT) of the ER (‘equal rates’) and ARD (‘all different rates’) models to select the optimal model for ancestral state reconstruction.

Figure S1 - Scanning electron microscopy images of *Solenopsis saevissima*.

Figure S2 - Scanning electron microscopy images and frontal photograph of *Solenopsis invicta*.

Figure S3 - Analysis ancestral state reconstruction (ASR) for *Solenopsis saevissima* with the ARD model with the names of the tips.

Figure S4 - Analysis ancestral state reconstruction (ASR) for *Solenopsis invicta* with the ARD model with the names of the tips.
