# Supplementary material for: Fire ants: What do rural and urban areas show us about occurrence, diversity, and ancestral state reconstruction?
Source: Genet Mol Biol. 2022 Mar 7;45(1):e20210120. doi: 10.1590/1678-4685-GMB-2021-0120 (PMC8932086; doi:10.1590/1678-4685-GMB-2021-0120)
Supplement: Table S1 - [file 1415-4757-GMB-45-1-e20210120-s1.pdf]

Genetics and Molecular Biology
https://doi.org/10.1590/1678-4685-GMB-2021-0120

Supplementary Material to "Fire ants: What do rural and urban areas show us about occurrence, diversity, and ancestral state reconstruction?"

Table S1 - Samples of *Solenopsis saevissima* and *Solenopsis invicta* collected in the present study. General characterization of nests, collection sites, and similarity percentage of specimens from each nest of *Solenopsis* with haplotypes deposited in the GenBank. (see below).

Nest		Specie	Area (rural or urban area)	GenBank			Geographic cordinate	
Number of nest	Sampling Point			Haplotype	Similarity (%)	Number of Accession	Latitude	Longitude
1	N1P1L1	*Solenopsis invicta*	Urban	H13	100%	AY950736.1	-23,529	-45,837
2	N2P1L1	*Solenopsis invicta*	Urban	H13	100%	AY950736.1	-23,529	-45,837
3	N4P1L1	*Solenopsis invicta*	Urban	H13	99%	AY950736.1	-23,529	-45,836
4	N5P2L1	*Solenopsis invicta*	Urban	H11	100%	AY950742.1 / AY950735.1	-23,525	-45,850
5	N6P2L1	*Solenopsis saevissima*	Urban	H4	100%	FJ467540.1	-23,524	-45,850
6	N7P3L1	*Solenopsis invicta*	Urban	H13	100%	AY950736.1	-23,527	-45,858
7	N9P4L1	*Solenopsis saevissima*	Urban	H24	99%	FJ467540.1	-23,538	-45,854
8	N10P4L1	*Solenopsis saevissima*	Urban	H4	99%	FJ467540.1	-23,538	-45,854
9	N11P4L1	*Solenopsis invicta*	Urban	H13	100%	AY950736.1	-23,539	-45,853
10	N12P5L1	*Solenopsis invicta*	Urban	H13	100%	AY950736.1	-23,532	-45,846
11	N13P5L1	*Solenopsis saevissima*	Urban	H23	98%	FJ467550.1 / FJ467540.1	-23,534	-45,845
12	N1P1L3	*Solenopsis invicta*	Urban	H13	100%	AY950736.1	-23,516	-46,184
13	N2P1L3	*Solenopsis invicta*	Urban	H11	99%	AY950742.1 / AY950735.1	-23,517	-46,185
14	N3P1L3	*Solenopsis saevissima*	Urban	H4	99%	FJ467540.1	-23,518	-46,185
15	N4P1L3	*Solenopsis invicta*	Urban	H13	99%	AY950736.1	-23,518	-46,186
16	N5P1L3	*Solenopsis saevissima*	Urban	H4	99%	FJ467540.1	-23,519	-46,187
17	N2P2L3	*Solenopsis invicta*	Urban	H13	99%	AY950736.1	-23,513	-46,152
18	N3P2L3	*Solenopsis invicta*	Urban	H13	99%	AY950736.1	-23,511	-46,151
19	N4P2L3	*Solenopsis invicta*	Urban	H13	99%	AY950736.1	-23,511	-46,152

Nest		Specie	Area (rural or urban area)	GenBank			Geographic cordinate	
Number of nest	Sampling Point			Haplotype	Similarity (%)	Number of Accession	Latitude	Longitude
20	N2P3L3	*Solenopsis invicta*	Urban	H20	99%	AY950736.1	-23,499	-46,178
21	N3P3L3	*Solenopsis invicta*	Urban	H13	100%	AY950736.1	-23,496	-46,177
22	N4P3L3	*Solenopsis invicta*	Urban	H13	99%	AY950736.1	-23,494	-46,181
23	N5P3L3	*Solenopsis invicta*	Urban	H13	99%	AY950736.1	-23,496	-46,182
24	N2P4L3	*Solenopsis saevissima*	Urban	H4	98%	FJ467540.1	-23,534	-46,205
25	N3P4L3	*Solenopsis saevissima*	Urban	H4	99%	FJ467540.1	-23,534	-46,205
26	N4P4L3	*Solenopsis invicta*	Urban	H11	99%	AY950742.1 / AY950735.1	-23,535	-46,203
27	N1P5L3	*Solenopsis saevissima*	Urban	H16	99%	FJ467540.1	-23,512	-46,196
28	N2P5L3	*Solenopsis saevissima*	Urban	H16	99%	FJ467540.1	-23,513	-46,194
29	N3P5L3	*Solenopsis invicta*	Urban	H11	99%	AY950742.1 / AY950735.1	-23,514	-46,195
30	N1P1L4	*Solenopsis saevissima*	Urban	H4	99%	FJ467540.1	-23,568	-46,048
31	N2P1L4	*Solenopsis saevissima*	Urban	H16	99%	FJ467540.1	-23,569	-46,046
32	N3P1L4	*Solenopsis saevissima*	Urban	H16	98%	FJ467540.1	-23,570	-46,047
33	N4P1L4	*Solenopsis invicta*	Urban	H13	99%	AY950736.1	-23,570	-46,048
34	N5P1L4	*Solenopsis invicta*	Urban	H13	99%	AY950736.1	-23,571	-46,047
35	N1P2L4	*Solenopsis invicta*	Urban	H13	100%	AY950736.1	-23,567	-46,035
36	N3P2L4	*Solenopsis invicta*	Urban	H13	100%	AY950736.1	-23,564	-46,033
37	N4P2L4	*Solenopsis invicta*	Urban	H13	99%	AY950736.1	-23,565	-46,032
38	N5P2L4	*Solenopsis invicta*	Urban	H13	99%	AY950736.1	-23,567	-46,033
39	N1P3L4	*Solenopsis saevissima*	Urban	H16	99%	FJ467540.1	-23,572	-46,034
40	N2P3L4	*Solenopsis invicta*	Urban	H13	100%	AY950736.1	-23,573	-46,035
41	N3P3L4	*Solenopsis invicta*	Urban	H13	99%	AY950736.1	-23,574	-46,032
42	N4P3L4	*Solenopsis invicta*	Urban	H13	100%	AY950736.1	-23,576	-46,027
43	N1PXL1	*Solenopsis saevissima*	Urban	H16	99%	FJ467540.1	-23,538	-45,851
44	N2PXL1	*Solenopsis saevissima*	Urban	H4	99%	FJ467540.1	-23,540	-45,850
45	N4PXL1	*Solenopsis saevissima*	Urban	H16	98%	FJ467540.1	-23,539	-45,851
46	N5PXL1	*Solenopsis invicta*	Urban	H13	100%	AY950736.1	-23,535	-45,852

Nest		Specie	Area (rural or urban area)	GenBank			Geographic cordinate	
Number of nest	Sampling Point			Haplotype	Similarity (%)	Number of Accession	Latitude	Longitude
47	N1PXIIL1	*Solenopsis invicta*	Urban	H13	100%	AY950736.1	-23,530	-45,843
48	N2PXIIL1	*Solenopsis saevissima*	Urban	H16	99%	FJ467540.1	-23,530	-45,843
49	N3PXIIL1	*Solenopsis invicta*	Urban	H13	100%	AY950736.1	-23,531	-45,843
50	N4PXIIL1	*Solenopsis saevissima*	Urban	H8	99%	FJ467540.1	-23,531	-45,841
51	N5PXIIL1	*Solenopsis saevissima*	Urban	H7	99%	FJ467540.1	-23,531	-45,841
52	N1PXIL1	*Solenopsis saevissima*	Urban	H16	99%	FJ467540.1	-23,534	-45,849
53	N2PXIL1	*Solenopsis saevissima*	Urban	H4	99%	FJ467540.1	-23,535	-45,846
54	N4PXIL1	*Solenopsis saevissima*	Urban	H16	99%	FJ467540.1	-23,532	-45,847
55	N1PXL3	*Solenopsis invicta*	Urban	H11	99%	AY950742.1 / AY950735.1	-23,531	-46,175
56	N2PXL3	*Solenopsis invicta*	Urban	H13	100%	AY950736.1	-23,515	-46,183
57	N3PXL3	*Solenopsis invicta*	Urban	H11	99%	AY950742.1 / AY950735.1	-23,515	-46,182
58	N4PXL3	*Solenopsis invicta*	Urban	H13	100%	AY950736.1	-23,515	-46,184
59	N5PXL3	*Solenopsis saevissima*	Urban	H16	99%	FJ467540.1	-23,514	-46,183
60	N1P4L3	*Solenopsis saevissima*	Urban	H4	99%	FJ467540.1	-23,536	-46,204
61	N4P5L3	*Solenopsis saevissima*	Urban	H16	98%	FJ467540.1	-23,517	-46,198
62	N7PXL3	*Solenopsis invicta*	Urban	H12	100%	EU352608.1 / HQ215540.1 / EF620556.1	-23,515	-46,182
63	N1NBL4	*Solenopsis saevissima*	Urban	H4	99%	FJ467540.1	-23,562	-46,051
64	N2NBL4	*Solenopsis invicta*	Urban	H13	99%	AY950736.1	-23,568	-46,053
65	N3NBL4	*Solenopsis saevissima*	Urban	H16	98%	FJ467540.1	-23,572	-46,049
66	N4NBL4	*Solenopsis invicta*	Urban	H13	99%	AY950736.1	-23,567	-46,063
67	N5NBL4	*Solenopsis invicta*	Urban	H13	100%	AY950736.1	-23,569	-46,053
68	N6NBL4	*Solenopsis saevissima*	Urban	H4	99%	FJ467540.1	-23,570	-46,053
69	N7NBL4	*Solenopsis invicta*	Urban	H13	100%	AY950736.1	-23,571	-46,054
70	N8NBL4	*Solenopsis saevissima*	Urban	H16	98%	FJ467540.1	-23,572	-46,053
71	N9NBL4	*Solenopsis invicta*	Urban	H13	100%	AY950736.1	-23,573	-46,057
72	N10NBL4	*Solenopsis invicta*	Urban	H13	99%	AY950736.1	-23,569	-46,058

Nest		Specie	Area (rural or urban area)	GenBank			Geographic cordinate	
Number of nest	Sampling Point			Haplotype	Similarity (%)	Number of Accession	Latitude	Longitude
73	N1CAL4	*Solenopsis invicta*	Urban	H13	99%	AY950736.1	-23,584	-46,041
74	N2CAL4	*Solenopsis invicta*	Urban	H13	99%	AY950736.1	-23,585	-46,040
75	N3CAL4	*Solenopsis invicta*	Urban	H21	99%	AY950736.1	-23,585	-46,039
76	N4CAL4	*Solenopsis invicta*	Urban	H13	99%	AY950736.1	-23,585	-46,040
77	N5CAL4	*Solenopsis invicta*	Urban	H11	99%	AY950742.1 / AY950735.1	-23,586	-46,042
78	N6CAL4	*Solenopsis invicta*	Urban	H13	99%	AY950736.1	-23,589	-46,045
79	N7CAL4	*Solenopsis invicta*	Urban	H22	99%	AY950736.1	-23,589	-46,046
80	N8CAL4	*Solenopsis invicta*	Urban	H13	99%	AY950736.1	-23,588	-46,045
81	N9CAL4	*Solenopsis saevissima*	Urban	H16	98%	FJ467540.1	-23,590	-46,041
82	N10CAL4	*Solenopsis invicta*	Urban	H13	99%	AY950736.1	-23,590	-46,044
83	N1BCL3	*Solenopsis saevissima*	Urban	H16	99%	FJ467540.1	-23,537	-46,219
84	N6BCL3	*Solenopsis invicta*	Urban	H13	99%	AY950736.1	-23,540	-46,229
85	N7BCL3	*Solenopsis invicta*	Urban	H13	99%	AY950736.1	-23,546	-46,222
86	N8BCL3	*Solenopsis invicta*	Urban	H13	99%	AY950736.1	-23,546	-46,222
87	N9BCL3	*Solenopsis saevissima*	Urban	H4	98%	FJ467540.1	-23,540	-46,229
88	N1JPL3	*Solenopsis invicta*	Urban	H13	99%	AY950736.1	-23,543	-46,258
89	N2JPL3	*Solenopsis invicta*	Urban	H11	99%	AY950742.1 / AY950735.1	-23,543	-46,258
90	N4JPL3	*Solenopsis saevissima*	Urban	H4	98%	FJ467540.1	-23,548	-46,258
91	N5JPL3	*Solenopsis invicta*	Urban	H13	100%	AY950736.1	-23,549	-46,257
92	N6JPL3	*Solenopsis saevissima*	Urban	H16	99%	FJ467540.1	-23,550	-46,250
93	N7JPL3	*Solenopsis invicta*	Urban	H11	99%	AY950742.1 / AY950735.1	-23,551	-46,250
94	SO1 N1	*Solenopsis saevissima*	Rural	H7	99%	FJ467540.1	-23,628	-46,110
95	SO1 N2	*Solenopsis saevissima*	Rural	H8	99%	FJ467540.1	-23,628	-46,110
96	SO1 N3	*Solenopsis saevissima*	Rural	H4	99%	FJ467540.1	-23,628	-46,110
97	SO1 N4	*Solenopsis saevissima*	Rural	H7	99%	FJ467540.1	-23,628	-46,110
98	SO1 N5	*Solenopsis saevissima*	Rural	H4	99%	FJ467540.1	-23,628	-46,110
99	SO1 N6	*Solenopsis saevissima*	Rural	H7	99%	FJ467540.1	-23,627	-46,110

Nest		Specie	Area (rural or urban area)	GenBank			Geographic cordinate	
**Number of nest**	**Sampling Point**			**Haplotype**	**Similarity (%)**	**Number of Accession**	**Latitude**	**Longitude**
100	SO1 N7	*Solenopsis saevissima*	Rural	H9	98%	FJ467550.1 / FJ467540.1	-23,627	-46,110
101	SO1 N8	*Solenopsis saevissima*	Rural	H4	99%	FJ467540.1	-23,627	-46,110
102	SO3 N3	*Solenopsis saevissima*	Rural	H9	98%	FJ467550.1 / FJ467540.1	-23,628	-46,135
103	SO3 N4	*Solenopsis saevissima*	Rural	H4	99%	FJ467540.1	-23,628	-46,135
104	SO3 N5	*Solenopsis saevissima*	Rural	H7	99%	FJ467540.1	-23,628	-46,135
105	SO3 N6	*Solenopsis saevissima*	Rural	H4	99%	FJ467540.1	-23,628	-46,135
106	SO3 N7	*Solenopsis saevissima*	Rural	H3	98%	FJ467540.1	-23,628	-46,135
107	SO3 N8	*Solenopsis saevissima*	Rural	H4	99%	FJ467540.1	-23,629	-46,135
108	AM1 N1	*Solenopsis saevissima*	Rural	H1	98%	FJ467540.1	-23,493	-46,197
109	AM1 N2	*Solenopsis saevissima*	Rural	H2	98%	FJ467540.1	-23,494	-46,198
110	AM1 N3	*Solenopsis saevissima*	Rural	H3	98%	FJ467540.1	-23,497	-46,199
111	AM1 N4	*Solenopsis saevissima*	Rural	H4	99%	FJ467540.1	-23,497	-46,199
112	AM1 N5	*Solenopsis saevissima*	Rural	H3	98%	FJ467540.1	-23,494	-46,200
113	AM1 N6	*Solenopsis saevissima*	Rural	H5	98%	FJ467540.1	-23,499	-46,203
114	AM1 N7	*Solenopsis saevissima*	Rural	H6	99%	FJ467540.1	-23,499	-46,199
115	AM1 N8	*Solenopsis saevissima*	Rural	H4	98%	FJ467540.1	-23,500	-46,198
116	E.N N1/P5	*Solenopsis saevissima*	Rural	H4	99%	FJ467540.1	-23,596	-46,147
117	E.V N1/P2	*Solenopsis saevissima*	Rural	H4	99%	FJ467540.1	-23,573	-46,255
118	E.B.F N1/P1	*Solenopsis saevissima*	Rural	H7	99%	FJ467540.1	-23,481	-46,151
119	E.B.F N1/P2	*Solenopsis saevissima*	Rural	H4	98%	FJ467540.1	-23,488	-46,147
120	E.T N5	*Solenopsis saevissima*	Rural	H4	99%	FJ467540.1	-23,426	-46,233
121	AM2 N1	*Solenopsis saevissima*	Rural	H7	99%	FJ467540.1	-23,578	-45,973
122	AM2 N2	*Solenopsis saevissima*	Rural	H14	98%	FJ467540.1	-23,578	-45,974
123	AM2 N3	*Solenopsis saevissima*	Rural	H4	98%	FJ467540.1	-23,580	-45,973
124	AM2 N4	*Solenopsis saevissima*	Rural	H8	98%	FJ467540.1	-23,581	-45,973
125	AM2 N5	*Solenopsis saevissima*	Rural	H9	98%	FJ467540.1	-23,582	-45,973
126	AM2 N6	*Solenopsis saevissima*	Rural	H7	98%	FJ467540.1	-23,583	-45,973

Nest		Specie	Area (rural or urban area)	GenBank			Geographic cordinate	
Number of nest	Sampling Point			Haplotype	Similarity (%)	Number of Accession	Latitude	Longitude
127	AM2 N7	*Solenopsis saevissima*	Rural	H15	98%	FJ467540.1	-23,585	-45,973
128	SC1 N5	*Solenopsis saevissima*	Rural	H4	98%	FJ467540.1	-23,554	-45,987
129	SC1 N6	*Solenopsis saevissima*	Rural	H16	99%	FJ467540.1	-23,554	-45,987
130	SC2 N1	*Solenopsis saevissima*	Rural	H9	98%	FJ467550.1 / FJ467540.1	-23,540	-46,101
131	SC2 N2	*Solenopsis saevissima*	Rural	H4	99%	FJ467540.1	-23,540	-46,101
132	SC2 N5	*Solenopsis saevissima*	Rural	H4	99%	FJ467540.1	-23,540	-46,100
133	SC2 N7	*Solenopsis saevissima*	Rural	H4	99%	FJ467540.1	-23,540	-46,099
134	SC3 N4	*Solenopsis saevissima*	Rural	H9	98%	FJ467550.1 / FJ467540.1	-23,545	-46,005
135	SC3 N5	*Solenopsis saevissima*	Rural	H4	99%	FJ467540.1	-23,546	-46,005
136	E.R.A N1/P4	*Solenopsis saevissima*	Rural	H8	99%	FJ467540.1	-23,539	-46,086
137	E.C.G N1/P3	*Solenopsis saevissima*	Rural	H9	98%	FJ467550.1 / FJ467540.1	-23,581	-46,015
138	E.PC N1/P2	*Solenopsis saevissima*	Rural	H9	98%	FJ467550.1 / FJ467540.1	-23,547	-46,038
139	E.S.G N1/P5	*Solenopsis saevissima*	Rural	H8	99%	FJ467540.1	-23,600	-46,065
140	SO2 N1	*Solenopsis saevissima*	Rural	H4	99%	FJ467540.1	-23,537	-45,850
141	SO2 N2	*Solenopsis saevissima*	Rural	H4	99%	FJ467540.1	-23,536	-45,850
142	SO2 N3	*Solenopsis saevissima*	Rural	H7	99%	FJ467540.1	-23,538	-45,850
143	SO2 N4	*Solenopsis saevissima*	Rural	H16	99%	FJ467540.1	-23,538	-45,850
144	SO2 N6	*Solenopsis saevissima*	Rural	H4	99%	FJ467540.1	-23,538	-45,850
145	SO2 N7	*Solenopsis saevissima*	Rural	H9	98%	FJ467550.1 / FJ467540.1	-23,638	-45,850
146	SO2 N8	*Solenopsis saevissima*	Rural	H4	99%	FJ467540.1	-23,638	-45,849
147	AM3 N1	*Solenopsis saevissima*	Rural	H9	98%	FJ467540.1	-23,638	-45,835
148	AM3 N2	*Solenopsis saevissima*	Rural	H19	98%	FJ467540.1	-23,637	-45,836
149	AM3 N3	*Solenopsis saevissima*	Rural	H4	98%	FJ467540.1	-23,637	-45,834
150	AM3 N4	*Solenopsis saevissima*	Rural	H4	99%	FJ467540.1	-23,637	-45,836
151	AM3 N5	*Solenopsis saevissima*	Rural	H9	98%	FJ467540.1	-23,637	-45,837
152	AM3 N6	*Solenopsis saevissima*	Rural	H9	98%	FJ467540.1	-23,637	-45,838
153	AM3 N7	*Solenopsis saevissima*	Rural	H9	99%	FJ467540.1	-23,637	-45,838

Nest		Specie	Area (rural or urban area)	GenBank			Geographic cordinate	
Number of nest	Sampling Point			Haplotype	Similarity (%)	Number of Accession	Latitude	Longitude
154	AM3 N8	*Solenopsis saevissima*	Rural	H4	98%	FJ467540.1	-23,637	-45,839
155	E.P N1/P2	*Solenopsis saevissima*	Rural	H7	99%	FJ467540.1	-23,538	-45,816
156	E.P N1/P3	*Solenopsis saevissima*	Rural	H4	99%	FJ467540.1	-23,538	-45,816
157	E.V.B N1/P2	*Solenopsis saevissima*	Rural	H4	99%	FJ467540.1	-23,511	-45,858
158	E.B.C N1/P5	*Solenopsis saevissima*	Rural	H4	99%	FJ467540.1	-23,544	-45,886
159	E.B.C N1/P6	*Solenopsis saevissima*	Rural	H9	98%	FJ467550.1 / FJ467540.1	-23,546	-45,892
160	E.B.R N1/P2	*Solenopsis saevissima*	Rural	H4	99%	FJ467540.1	-23,522	-45,840
161	SO3 N2	*Solenopsis invicta*	Rural	H10	99%	AY950751.1/ AY950742.1 / AY950735.1	-23,629	-46,134
162	SO3 N1	*Solenopsis saevissima*	Rural	H9	98%	FJ467550.1 / FJ467540.1	-23,629	-46,134
163	E.N N1/P6	*Solenopsis invicta*	Rural	H11	99%	AY950742.1 / AY950735.1	-23,601	-46,160
164	E.V N1/P3	*Solenopsis invicta*	Rural	H12	99%	EU352608.1 / HQ215540.1 / EF620556.1	-23,586	-46,256
165	E.T N4	*Solenopsis invicta*	Rural	H13	100%	AY950736.1	-23,426	-46,233
166	SC1 N1	*Solenopsis invicta*	Rural	H11	99%	AY950752.1/ AY950742.1/ AY950735.1/ AY950732.1	-23,555	-45,988
167	SC1 N2	*Solenopsis invicta*	Rural	H13	100%	AY950736.1	-23,555	-45,987
168	SC1 N3	*Solenopsis invicta*	Rural	H13	100%	AY950736.1	-23,555	-45,987
169	SC1 N4	*Solenopsis invicta*	Rural	H13	100%	AY950736.1	-23,554	-45,987
170	SC1N7	*Solenopsis invicta*	Rural	H13	100%	AY950736.1	-23,555	-45,988
171	SC1N8	*Solenopsis invicta*	Rural	H11	99%	AY950752.1/ AY950742.1/ AY950735.1/ AY950732.1	-23,554	-45,987
172	SC2 N3	*Solenopsis invicta*	Rural	H11	99%	AY950752.1/ AY950742.1/ AY950735.1/ AY950732.1	-23,540	-46,100
173	SC2 N4	*Solenopsis invicta*	Rural	H11	99%	AY950752.1/ AY950742.1/ AY950735.1/ AY950732.1	-23,540	-46,100
174	SC2 N6	*Solenopsis invicta*	Rural	H13	100%	AY950736.1	-23,540	-46,100
175	SC2 N8	*Solenopsis invicta*	Rural	H11	99%	AY950742.1 / AY950735.1	-23,540	-46,100
176	SC3 N1	*Solenopsis invicta*	Rural	H13	100%	AY950736.1	-23,545	-46,006

Nest		Specie	Area (rural or urban area)	GenBank			Geographic cordinate	
Number of nest	Sampling Point			Haplotype	Similarity (%)	Number of Accession	Latitude	Longitude
177	SC3 N2	*Solenopsis invicta*	Rural	H13	100%	AY950736.1	-23,545	-46,006
178	SC3 N3	*Solenopsis invicta*	Rural	H13	100%	AY950736.1	-23,545	-46,006
179	SC3 N6	*Solenopsis invicta*	Rural	H17	100%	AY950736.1	-23,546	-46,005
180	SC3 N7	*Solenopsis invicta*	Rural	H11	99%	AY950752.1/ AY950742.1/ AY950735.1/ AY950732.1	-23,546	-46,005
181	E. R. A N1/P5	*Solenopsis invicta*	Rural	H10	99%	AY950752.1/ AY950742.1/ AY950735.1/ AY950732.1	-23,534	-46,079
182	E.C.G N1/P2	*Solenopsis invicta*	Rural	H13	100%	AY950736.1	-23,569	-46,016
183	E.PC N1/P1	*Solenopsis invicta*	Rural	H18	100%	AY950736.1	-23,552	-46,041
184	E.S.G N1/P6	*Solenopsis invicta*	Rural	H13	100%	AY950736.1	-23,600	-46,052
185	E.V.B N1/P3	*Solenopsis invicta*	Rural	H13	100%	AY950736.1	-23,508	-45,854
186	E.B.R N1/P1	*Solenopsis invicta*	Rural	H11	99%	AY950742.1 / AY950735.1	-23,527	-45,844
