# Supplementary material for: Fire ants: What do rural and urban areas show us about occurrence, diversity, and ancestral state reconstruction?
Source: Genet Mol Biol. 2022 Mar 7;45(1):e20210120. doi: 10.1590/1678-4685-GMB-2021-0120 (PMC8932086; doi:10.1590/1678-4685-GMB-2021-0120)
Supplement: Table S2 - [file 1415-4757-GMB-45-1-e20210120-s2.pdf]

Genetics and Molecular Biology
https://doi.org/10.1590/1678-4685-GMB-2021-0120

Supplementary Material to "Fire ants: What do rural and urban areas show us about occurrence, diversity, and ancestral state reconstruction?"

Table S2 - Likelihood ratio test (LRT) of the ER ('equal rates') and ARD ('all different rates') models to select the optimal model for ancestral state reconstruction. Note that the ARD model is highlighted in bold because better explained our data for both species in this study.

	Solenopsis saevissima	*Solenopsis invicta*
Model	Likelihood Ratio Test (LRT)	
ER	-60.087928	-51.160499
ARD	**-59.924126**	**-48.835912**
