# Supplementary material for: Fire ants: What do rural and urban areas show us about occurrence, diversity, and ancestral state reconstruction?
Source: Genet Mol Biol. 2022 Mar 7;45(1):e20210120. doi: 10.1590/1678-4685-GMB-2021-0120 (PMC8932086; doi:10.1590/1678-4685-GMB-2021-0120)
Supplement: Figure S1 - [file 1415-4757-GMB-45-1-e20210120-s3.pdf]

Genetics and Molecular Biology
https://doi.org/10.1590/1678-4685-GMB-2021-0120

Supplementary Material to "Fire ants: What do rural and urban areas show us about occurrence, diversity, and ancestral state reconstruction?"

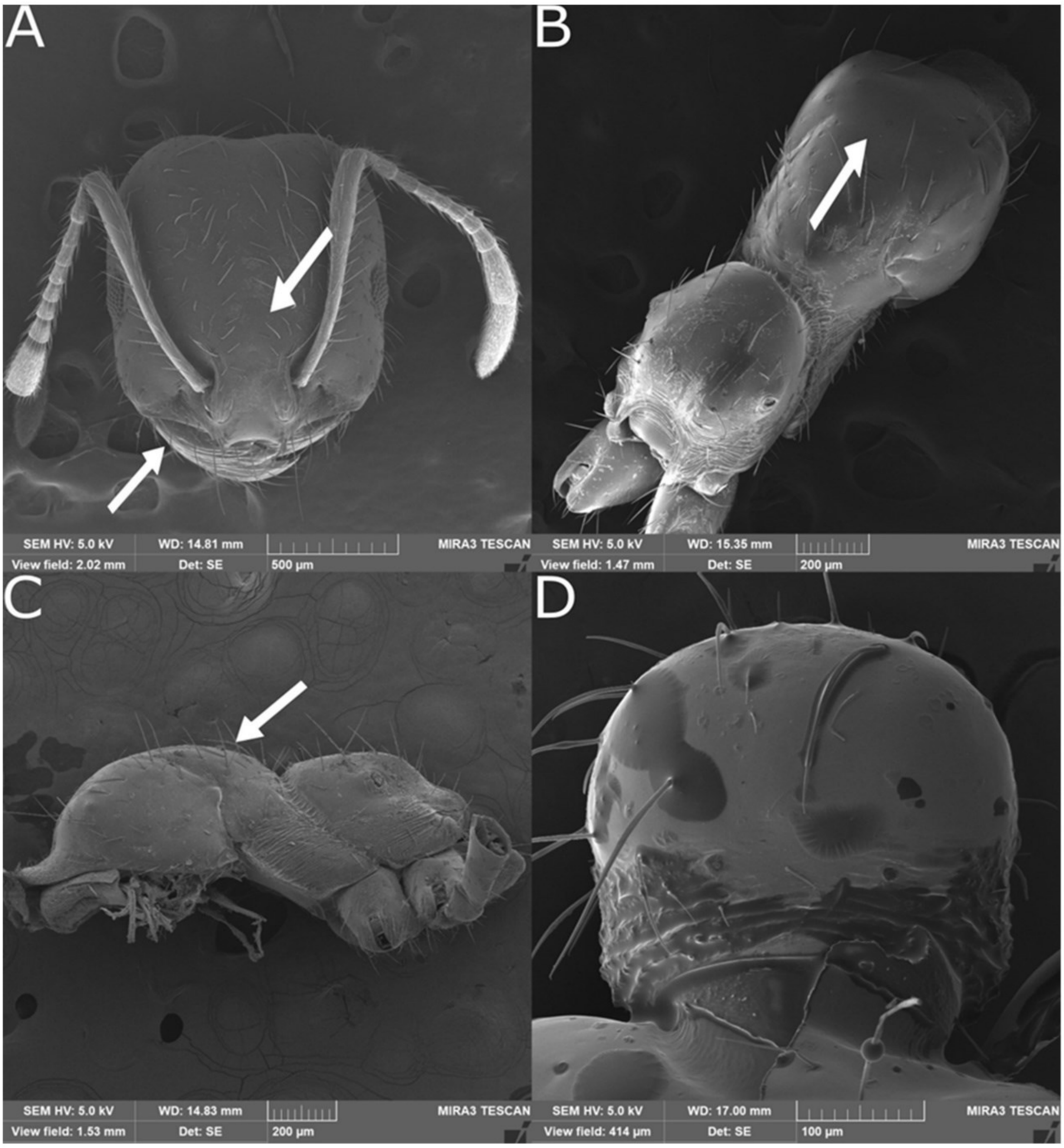

Figure S1 - Scanning electron microscopy images of *Solenopsis saevissima*. **A.** Head in frontal view indicating where the median streak is not seen and the complete mandibular costulae. **B.** Mesosome in dorsal view indicating the weakest promesonotum suture. **C.** Mesosome in lateral view; arrow indicates mesonotum slightly convex. **D.** Postpetiole higher than long in posterior view, with transversely rugose sculpting only in the lower portion, and smooth in the upper surface.
