# Supplementary material for: Fire ants: What do rural and urban areas show us about occurrence, diversity, and ancestral state reconstruction?
Source: Genet Mol Biol. 2022 Mar 7;45(1):e20210120. doi: 10.1590/1678-4685-GMB-2021-0120 (PMC8932086; doi:10.1590/1678-4685-GMB-2021-0120)
Supplement: Figure S2 - [file 1415-4757-GMB-45-1-e20210120-s4.pdf]

Genetics and Molecular Biology
https://doi.org/10.1590/1678-4685-GMB-2021-0120

Supplementary Material to "Fire ants: What do rural and urban areas show us about occurrence, diversity, and ancestral state reconstruction?"

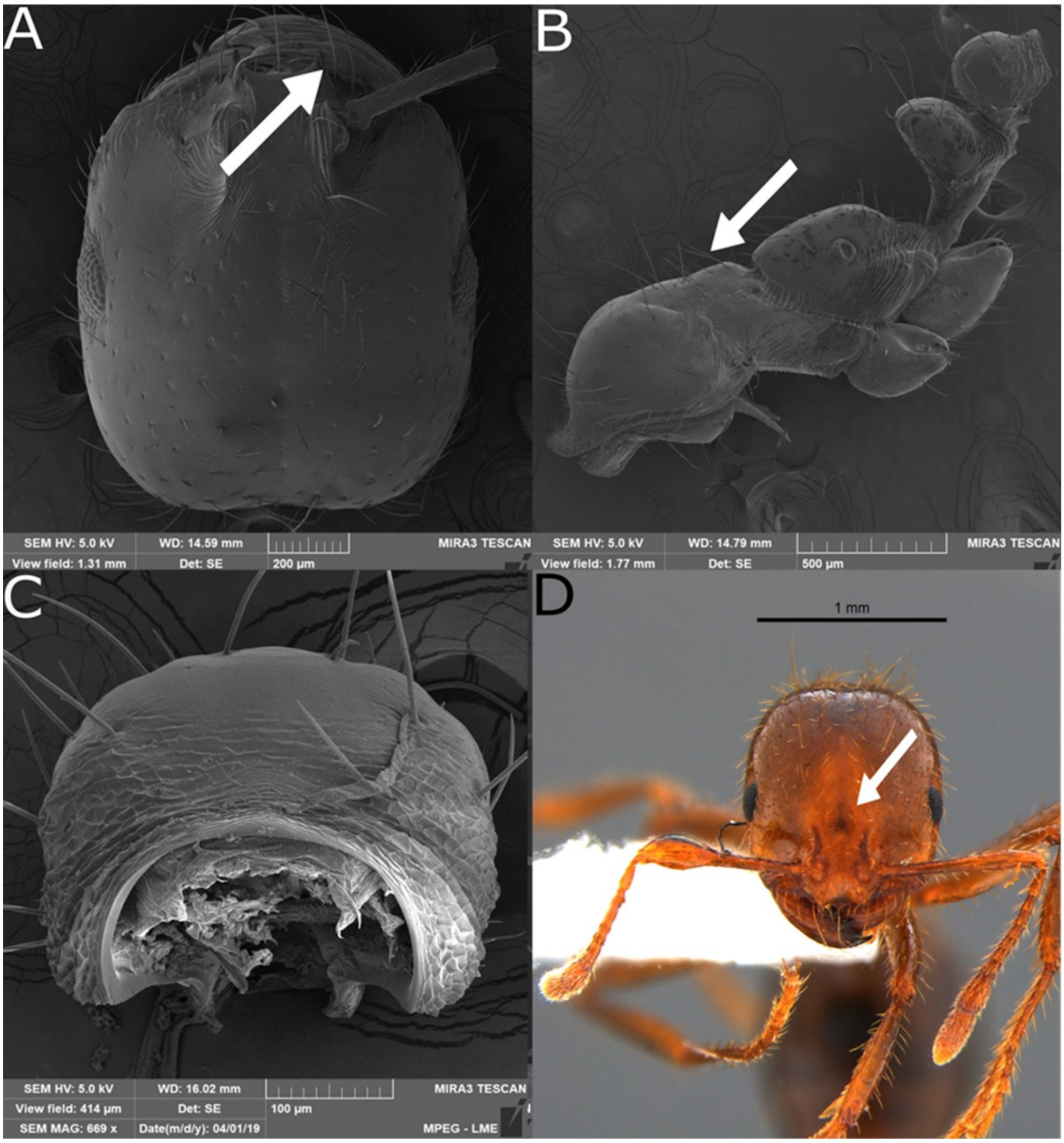

Figure S2 - Scanning electron microscopy images and frontal photograph of *Solenopsis invicta*. **A.** Head in frontal view indicating the mandibular costulae absent medially. **B.** Mesosome in lateral view, arrow indicating the convex mesonotum. **C.** Portion of post-petiole in posterior view wider than high with transversely rugose to punctate-rugose sculpting covering most of the view. **D.** Head in frontal view showing the median frontal streak.
