# Supplementary material for: Fire ants: What do rural and urban areas show us about occurrence, diversity, and ancestral state reconstruction?
Source: Genet Mol Biol. 2022 Mar 7;45(1):e20210120. doi: 10.1590/1678-4685-GMB-2021-0120 (PMC8932086; doi:10.1590/1678-4685-GMB-2021-0120)
Supplement: Figure S3 - [file 1415-4757-GMB-45-1-e20210120-s5.pdf]

Genetics and Molecular Biology
https://doi.org/10.1590/1678-4685-GMB-2021-0120

Supplementary Material to "Fire ants: What do rural and urban areas show us about occurrence, diversity, and ancestral state reconstruction?"

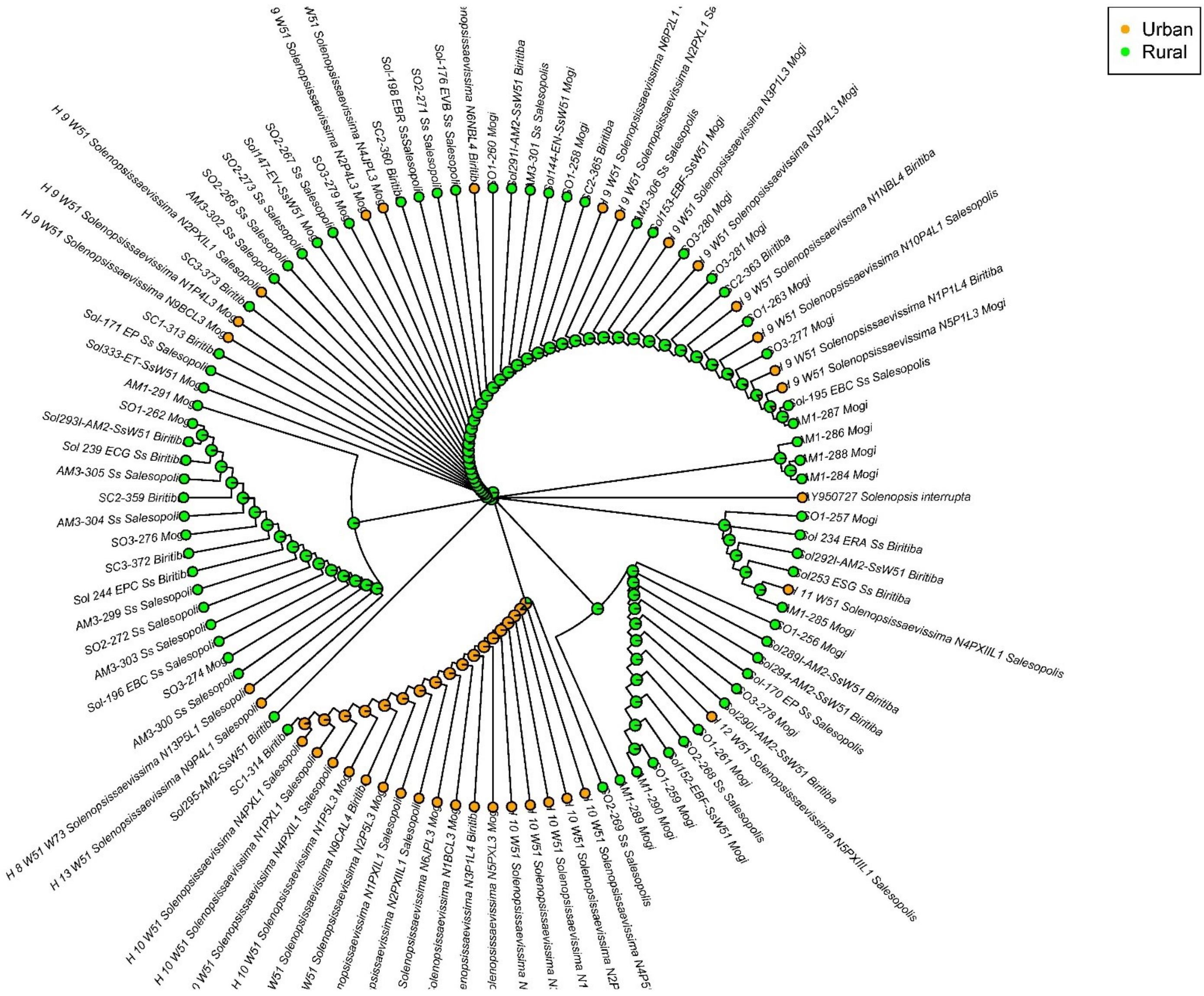

Figure S3 - Analysis ancestral state reconstruction (ASR) for *Solenopsis saevissima* with the ARD model with the names of the tips.
